# The clinical features and outcomes of Tolosa-Hunt syndrome

**DOI:** 10.1186/s12886-021-02007-0

**Published:** 2021-05-27

**Authors:** Hyuna Kim, Shin Yeop Oh

**Affiliations:** 1 Department of Ophthalmology, Soonchunhyang University Seoul Hospital, Soonchunhyang University College of Medicine, Seoul, Korea; 2 Department of Ophthalmology, Samsung Changwon Hospital, Sungkyunkwan University School of Medicine, 158, Paryong-ro, Masanhoewon-gu, 51353 Changwon, Korea

**Keywords:** Tolosa-Hunt syndrome, painful ophthalmoplegia, paralysis, orbital pain, optic nerve

## Abstract

**Background:**

The objective of this study was to investigate the clinical features and outcomes of Tolosa-Hunt syndrome (THS).

**Methods:**

A retrospective review of the medical records was performed on patients with THS between March 2016 and January 2020. A total of eleven patients fulfilling the International Classification of Headache Disorders (ICHD-3 beta) diagnostic criteria for THS were included in this study.

**Results:**

The average age of the patients with THS was 57.18 ± 15.32 years and the mean duration of recovery was 26.91 ± 24.35 days. All eleven patients had orbital or periorbital pain as the first symptom followed by diplopia. Ptosis was found in five patients (45.45 %) in the involved eye. Sixth cranial nerve (CN) palsy was most common (eight cases, 72.73 %), followed by third and fourth CN palsy (five cases, 45.45 %, respectively), optic neuritis (two cases, 18.18 %), and trigeminal nerve and facial nerve palsy (one case, 9.09 %, respectively). One patient with optic neuritis failed to recover visual acuity and the other ten patients completely recovered their ocular motor limitation. All patients were initially treated with steroids. One patient relapsed after five weeks and one patient had a history of THS five years earlier.

**Conclusions:**

THS responded well to steroid treatment, but if it was accompanied by optic neuritis as optic nerve involvement, we suggest follow-up with high dose steroid treatment especially was important.

## Introduction

Tolosa-Hunt syndrome (THS) is an uncommon idiopathic granulomatous inflammatory disease of the cavernous sinus, superior orbital fissure, or orbit associated with ocular pain and ophthalmoplegia [1, 2]. However, the etiology of THS is still unknown. Thus, the diagnosis of THS should exclude other causes of painful ophthalmoplegia such as tumors, vasculitis, meningitis, sarcoidosis, diabetic ophthalmoplegia, and pseudotumor [3, 4]. THS is described in the International Classification of Headache Disorders (ICHD 3-beta) diagnostic criteria as unilateral orbital pain associated with paresis of one or more of the third, fourth, and/or sixth cranial nerves caused by a granulomatous inflammation in the cavernous sinus, superior orbital fissure or orbit (Table 1) [5]. In clinical practice, the disorder is diagnosed based on the above diagnostic criteria and treatment with corticosteroids is generally recommended. There are relatively few reports on the clinical course of THS, so the objective of this retrospective study was to investigate the clinical outcomes of THS.

**Table 1:** ICHD-3 beta diagnostic criteria for 13.7 Tolosa-Hunt syndrome

A.	Unilateral headache fulfilling criterion C
B.	Both of the following: 1. Granulation inflammation of the cavernous sinus, superior orbital fissure or orbit, demonstrated by MRI or biopsy 2. Paresis of one or more of the ipsilateral third, fourth and/or sixth cranial nerves
C.	Evidence of causation demonstrated by both of the following: 1. Headache has preceded paresis of the third, fourth and/or sixth cranial nerves by ≤ 2weeks, or developed with it 2. Headache is localized around the ipsilateral brow and eye
D.	Not better accounted for by another ICHD-3 diagnosis.

ICHD 3 beta = International Classification of Headache Disorders, third edition

MRI = magnetic resonance imaging

## Methods

 The Institutional Review Board for Human studies at Samsung Changwon Hospital (Changwon, Republic of Korea) reviewed and approved this study protocol (IRB No. 2020-06-014). As this study was a retrospective study, informed consent was obtained from all patients before the imaging examination and all identifiable personal information of patients would be anonymized. All study conduct adhered to the tenets of the Declaration of Helsinki for the use of human participants in biomedical research. The medical records of patients diagnosed with THS from March 2016 to January 2020 at Samsung Changwon Hospital were retrospectively reviewed. The diagnosis of THS was defined by (1) unilateral headache or orbital pain; (2) headache preceded with paresis of the third cranial nerve (CN), fourth CN, and/or sixth CN for two weeks, or developed with it; and (3) headache localized around the ipsilateral brow and eye based on ICHD-3 beta criteria [5]. All patients underwent brain or orbit magnetic resonance imaging (MRI) and enhancing lesions of the cavernous sinus, superior orbital fissure, and/or orbital apex were identified. Furthermore, other causes of painful ophthalmoplegia including tumors, vasculitis, basal meningitis, sarcoidosis, migraine, cranial herpes zoster, giant cell arteritis, myositis, pseudotumor, and thyroid ophthalmopathy were excluded. Patients who did not show confirmed recovery within a follow-up duration of less than six months were excluded. Case notes were reviewed to obtain data on patient demographics (age, sex), previous medical history (hypertension, diabetes mellitus, or cancer), symptoms (recorded as orbital pain, diplopia, ptosis, visual loss, and facial numbness), the involved cranial nerves, laboratory results, neuroimaging, the symptom-resolution interval, and treatment. All patients were given oral methylprednisolone (MPD) at 50 mg/day or intravenous (IV) MPD 1 g/day initially and then switched to a gradually decreased dose until the signs and symptoms resolved. The patients with previously diagnosed paralytic or restrictive strabismus, congenital strabismus, or with previous extraocular muscle surgery were excluded. At the first visit, the patients underwent ophthalmologic assessments including slit-lamp examination, intraocular pressure measurements, ocular motor limitation assessment, and fundus photography. In addition, the patients were examined the hematological tests and cerebrospinal studies. At the final follow-up visit, THS recovery was deemed complete or non-recovered based on ocular motor limitation and visual acuity. Complete recovery was defined as the absence of ocular motor limitation and other CN abnormalities and non-recovery was defined as sequelae of CN function.

## Results

A total of eleven patients (night males and two females) were included in this study and the mean follow-up time was 201.27 ± 31.36 days after the initial visit. The average age at THS onset was 57.18 ± 15.32 years (range, 35–78 years). All eleven patients had orbital or periorbital pain as the first symptom followed by diplopia or ptosis. Diplopia and ptosis were found in eleven patients (100 %) and five patients (45.45 %), respectively. Sixth CN palsy was most common (eight cases, 72.73 %), followed by fourth and sixth CN palsy (five cases, 45.45 %, respectively), optic nerve (ON) involvement (two cases, 18.18 %), and trigeminal nerve and facial nerve palsy (one case, 9.09 %, respectively). At the initial examination, four patients had one CN involved, three patients had two CNs involved, and two patients had three CNs involved. Complete recovery was seen in ten (90.91 %) patients and in one patient, the ocular motor limitation was completely recovered but visual acuity was not recovered. The mean duration of recovery was 26.91 ± 24.35 days (range, 8–73 days) (Table 2). The clinical summary and MRI findings of the patients are presented in Table 3. All patients were initially treated with steroid. Six patients received oral MPD at 50 mg/day and five patients received IV MPD at 1 g/day. The laboratory test results showed an elevated erythrocyte sedimentation rate (ESR) in three patients and no patients had elevated C-reactive protein (CRP) values. Three patients with elevated ESR had a paralysis of all third, fourth and sixth CNs. In two patients with optic neuritis as ON involvement, one had fourth CN palsy at the first visit with a history of facial nerve palsy before four months. ON swelling was observed three days later, and IV MPD at 1 g/day was administered. Another patient had third, fourth, and sixth CN palsy at the first visit and oral MPD at 50 mg/day was administered. After five weeks, decreased visual acuity was observed and IV MPD at 1 g/day was injected. The first patient recovered completely after treatment, but the second patient did not recover from visual impairment. Of all the patients, two patients relapsed, and one patient had recurrence of visual impairment at 5 weeks interval. The other patient had a history of treatment for THS five years prior according to the medical record and was included as a relapsed patient.

**Table 2:** Demographics of patients with Tolosa-Hunt syndrome

Parameters			
Total patients (n)	11		
Male : Female (n)	9 (81.12 %) : 2 (18.18 %)		
Age at time of onset (years)	57.18 ± 15.32 (range : 35–78)		
Involved eye (right : left)	5 (45.45 %) : 6 (54.55 %)		
Duration of follow-up (days)	201.27 ± 31.36		
Duration of recovery (days)	26.91 ± 24.35 (range : 8–73)		
Complete recovery state (n)	10 (90.91 %)		
ESR (mm/hour)	16.73 ± 13.76 (range : 2–52)		
CRP (mg/L)	1.15 ± 0.84 (range : 0.3–2.6)		
Diplopia (n)	11 (100 %)		
Periorbital pain (n)	11 (100 %)		
CN nerve involvement cases (n) Optic nerve Third CN Fourth CN Trigeminal nerve Sixth CN	2 (18.18 %) 5 (45.45 %) 5 (45.45 %) 1 (9.09 %) 8 (72.73 %)		
Ptosis (n)	5 (45.45 %)		
Hypertension (n)	3 (27.27 %)		
Diabetes mellitus (n)	2 (18.18 %)		
Recurrence state (n)	2 (28.18 %)		

Values are presented as mean ± SD

SD = standard deviation

ESR = erythrocyte sedimentation rate; CRP = C-reactive protein; CN = cranial nerve

**Table 3:** Clinical summary of patients with Tolosa-Hunt Syndrome

Case	Sex		Age	Clinical findings	Involved CN	MRI findings	ESR (0–20)			CRP (0–5)	Treatment (per day)	Relapse
1	M	73		Diplopia	6 CN	Enhanced left cavernous sinus and clinoid process		15	0.9		Oral MPD 50 mg	-
2	M	74		Diplopia, Ptosis	Optic nerve, 3/4/6 CN	Enlarged and enhanced left cavernous sinus/apex/optic nerve		52	2.6		Oral MPD 50 mg	5 weeks interval
3	M	52		Diplopia	Optic nerve, 4 CN	Mild thickening and enhanced right cavernous sinus/apex (superior/inferior orbital fissure)		10	2.5		IV MPD 1 g	-
4	F	46		Diplopia, Ptosis	3/4/6 CN	Enhanced focal triangular shaped right cavernous sinus with enlarged right superior ophthalmic vein		21	0.3		IV MPD 1 g	-
5	M	68		Diplopia, Ptosis	3 CN	Enhanced focal right superior orbital fissure		12	2.0		Oral MPD 50 mg	5 years ago
6	M	46		Diplopia	6 CN	Slightly bulging of right cavernous sinus		2	0.5		Oral MPD 50 mg	-
7	M	46		Diplopia	6 CN, facial nerve	Suspicious enhanced left superior orbital fissure		10	0.7		Oral MPD 50 mg	-
8	M	69		Diplopia	4 CN	Enhanced left cavernous sinus/superior orbital fissure		8	0.8		Oral MPD 50 mg	-
9	M	78		Diplopia, Ptosis	3/6 CN	Enlarged and enhanced left cavernous sinus		11	0.7		IV MPD 1 g	-
10	F	42		Diplopia, Ptosis	3/4/6 CN	Enhanced right cavernous sinus and extending to orbital apex		30	0.5		IV MPD 1 g	-
11	M	35		Diplopia	Trigeminal nerve, 6 CN	Enhanced left cavernous sinus and extracranial portion of mandibular division of trigeminal nerve		13	1.1		IV MPD 1 g	-

MRI = magnetic resonance imaging; ESR = erythrocyte sedimentation rate; CRP = C-reactive protein

CN = cranial nerve; MPD = methylprednisolone; IV = intravenous

## Discussion

This retrospective study of eleven cases of THS based on ICHD-3 beta criteria showed that the clinical manifestations were variable. The sixth CN (72.73 %) was most commonly involved, followed by the third (45.45 %), and fourth CN (45.45 %). Previous studies reported a high rate of third CN (78–91 %) involvement [6, 7], however, Curone et al. reported a low rate of third CN (39 %) involvement [8]. As such, CN involvement has been variably reported in each study. THS is usually treated with steroid, but there are no rules regarding drugs, formulations, routes of administration, or therapeutic doses. Steroid treatment generally includes prednisone, MPD, and dexamethasone given orally or by IV injection. In this study, five patients were treated with IV MPD at 1 g/day for three days, and six patients received oral MPD at 50 mg/day for seven days, then the dosage was tapered. The difference in treatment dose was determined in consideration of the patient’s age, the severity of their symptoms, and underlying diseases. After the initiation of MPD treatment, pain resolved within two days in all patients, and ocular motor limitation gradually improved. Other studies reported that most patients achieved pain relief within 72 hours and that CN palsy resolved over two to eight weeks [6, 7]. In the patients in this study, the recovery period varied from one to eight weeks, similar to the previous report. Steroid treatment relieves the pain quickly, but the signs do not resolve immediately after treatment. As in this study and previous studies, because steroid administration had dramatic effects, when THS is diagnosed, it is important to start steroid therapy immediately. An accurate diagnosis is important for quick and appropriate treatment, but many diseases must be considered in the differential diagnosis of painful ophthalmoplegia. Tolosa-Hunt syndrome is a diagnosis of exclusion requiring various examination and careful evaluation to rule out tumors, vascular causes, or other forms of inflammation in the lesion of the cavernous sinus and superior orbital fissure (Table 4). The role of MRI in the criteria has been included as “granulomatous inflammation demonstrated by MRI or biopsy” in the ICHD-3 beta criteria. In most studies and our study, no patient had a biopsy or pathological examination [3, 4, 6, 7]. Also, the location of lesion is an important factor in the differential diagnosis, especially an orbital pseudotumor with characteristics and steroid response similar to THS [9]. Therefore, MRI plays a key role in the diagnosis of THS. Since THS can be diagnosed by anatomical location, MRI should be performed if THS is suspected in a case of painful ophthalmoplegia. The recurrence rate in our study (18.18 %) was similar to that of Rui et al. (23 %) [6] and Colnaghi et al. (21 %) [3] but was lower than that reported by Zhang et al. (37 %) [7]. Two patients in this study experienced recurrences, one recurred after five weeks and the other had a history of THS diagnosis five years earlier. THS patients have a good prognosis but recurrences occur in about 21–50 % of the cases over an interval of months to years [3, 7, 10]. Thus, it is important to consider follow-up and the possibility of a recurrence after a full recovery. In this study, the laboratory test results showed elevated ESR values in three patients, which were the patients with all third, fourth, and sixth CN involvement. However, no patients with non-elevated ESR had paralysis of all third, fourth, and sixth CNs. In patients with elevated ESR, one patient did not recover from visual impairment, and the other two showed complete recovery, but the recovery period of the three patients was about 2 months, which was longer than the average recovery period of all patients. The longer recovery period can be considered in association with the clinical findings that there are many involved CN palsy, and the ESR values could be considered as a factor predicting the severity of the disease and the recovery period. However, it is expected that further studies of the blood test results in more patients will be needed in the future. Meanwhile, ON dysfunction has been reported, indicating that the pathological process may involve the orbital apex [10]. Two patients in this study had optic neuritis as ON involvement and enhanced apex lesions were confirmed by orbit MRI. One patient had fourth CN palsy and optic neuritis and recovered completely after steroid treatment. In contrast, in other patients with third, fourth, and sixth CN palsy, relapsed five weeks later with retrobulbar optic neuritis and third, fourth, and sixth CN palsy. After high-dose steroid treatment, the ocular motor limitation was completely recovered, but the vision loss (recognition of hand motion) did not improve and a pale optic disc was observed at follow-up. Kline and Hoyt [10] reported that the optic disc may be normal, swollen, or pale in appearance, with visual decline ranging from minimal to blindness, and variable final visual acuity. In the two cases in this study, the opposite results were found. In one case, the visual acuity was completely recovered and in the other case, it was not recovered despite the same treatment. In patients whose vision did not recover, oral MPD at 50 mg/day was prescribed at the initial examination because of old age and uncontrolled diabetic history. At recurrence of THS with optic neuritis, 1 g of MPD was administered, but the loss of vision did not recover. It is thought that the damaged ON axons could not be recovered due to persistent granulomatous inflammation. We suggest that initial IV MPD at 1 g/day might have a better prognosis considering the elevated ESR and extended inflammation of cavernous sinus and orbital apex. Although optic neuritis as ON involvement is not common in THS, the occurrence of ON dysfunction should be confirmed through a visual acuity examination during the follow-up period and immediate high dose steroid treatment should be initiated. In addition, even if the ocular motor limitation have improved, it is necessary to check for recurrence or ON function through sufficient follow-up. Our study had several limitations. First, it was a retrospective, single-hospital-based study, suggesting possible selection bias. Second, the sample size was small due to the relatively uncommon occurrence of the disease. Third, the follow-up intervals were not standardized and were not long-term. Nevertheless, this study showed the strength of analyzing eleven cases and reporting various THS clinical symptoms. In conclusion, considering that THS may cause optic neuritis as ON involvement, immediate high dose steroid treatment should be performed at the time of diagnosis. In addition, since there is a possibility of recurrence, long-term follow-up is necessary.

**Table 4:** Diagnostic evaluation of Tolosa-Hunt syndrome

A. Laboratory tests, Complete blood count, Serum chemistry (electrolyte, glucose, liver and renal function), Erythrocyte sedimentation rate (ESR), C-reactive protein (CPR), Hemoglobin A1C (HbA1C), Angiotensin converting enzyme (ACE), Thyroid function test, Auto-antibodies
B. Cerebrospinal fluid (CSF) studies
C. Neuro-radiological studies, Brain or Orbit MRI, Orbit CT, Cerebral angiography
D. Ophthalmological examinations, Visual acuity, Intraocular pressure (IOP), Slit lamp examination, Fundus examination, Extraocular muscle movement
E. Biopsy of cavernous sinus (in some cases)

MRI = magnetic resonance imaging; CT = computed tomography

## Data Availability

The dataset used and/or analysed during the current study are available from the corresponding author on reasonable request.
